# T131. INCIDENCE OF FIRST-EPISODE PSYCHOSIS IN THE CATCHMENT AREA OF RIBEIRÃO PRETO, BRAZIL

**DOI:** 10.1093/schbul/sby016.407

**Published:** 2018-04-01

**Authors:** Cristina Del-Ben, Shuhama Rosana, Camila Loureiro, Taciana Ragazzi, Daniela Zanatta, Silvia Tenan, Jair Ferreira-Santos, Paulo Louzada-Junior, Antonio dos Santos, Craig Morgan, Paulo Menezes

**Affiliations:** 1 Ribeirão Preto Medical School, University of São Paulo; 2 Institute of Psychiatry, Psychology & Neuroscience, King’s College London

## Abstract

**Background:**

In the study “Schizophrenia and Other Psychoses Translational Research: Environment and Molecular Biology” (STREAM), we estimated the incidence of first-episode psychosis (FEP) in the catchment area of Ribeirão Preto, Brazil, and investigated the role of environmental and biological factors in the aetiology of the psychoses. STREAM is part of the international consortium “European Network of National Schizophrenia Networks Studying Gene-Environment Interactions” (EU-GEI).1 Here, we describe the incidence of FEP in this Brazilian catchment area according to some demographic characteristics of the population.

**Methods:**

Twenty-six counties compose the catchment area under study. Ribeirão Preto is the main city of the region, with a population of 604,682 inhabitants, population density of 929.8 inhabitants/Km2, per capita gross intern product (GIP) of U$ 9,143,20 and human development index (HDI) of 0.800, ranked 40th among 5,570 Brazilian municipalities. The population of the remaining 25 counties varied from 1,953 to 110,074 inhabitants (median=23,862), median population density of 75.0 inhabitants/Km2 (interval 13.2–308.4 inhabitants/ Km2), median GIP of U$5,254,80 (interval U$2,234,80–U$20,747,40), and HDI values ranging from 145th to 2,282nd in the national ranking. The sample was composed by individuals aged 16 to 64 years old, and with a first contact with mental health services due to psychotic symptoms in a 3-year period (1st April 2012 – 31st March 2015). Statistical analyses were carried out using Stata 13 software. We used the estimated population during the 3-year period based on the 2010 Brazilian Census to calculate person-years at risk. Incidence rates were estimated by sex, age, self-reported skin colour, and city of living.

**Results:**

We identified 588 FEP patients over 3 years. The incidence rate of psychosis was higher in male (21.8, 95%CI=19.5–24.4) than female (18.1, 95%IC=16.1–20.5), non-white (20.9, 95%IC=18.1–23.9) than white patients (15.8, 95%IC=14.1–17.6), and those living in counties (22.3, 95%IC=20.1–24.5) other than the main city, Ribeirão Preto (17.3, 95%IC=15.2–19.6). The incidence rate of FEP declined with age.

**Discussion:**

The proportion of patients reporting themselves as white (52.1%) was lower than that described for the catchment area under study (66.3%), confirming the higher risk of psychosis among those from minority groups.2 The incidence rates of FEP observed in the Ribeirão Preto catchment area are roughly similar to those reported in São Paulo city3 and in a large city in Southern Italy4, but lower to those described in large urban centres in some European countries5, which converges to the heterogeneity of the incidence of psychosis worldwide. We found a lower incidence rate of FEP in the main city of the catchment area, in comparison with the remaining cities of the region, which have a lower population density, but worse socioeconomic indicators. These preliminary results suggest an effect of socioeconomically deprived contexts in the incidence of psychosis in Brazil, as described in developed countries.6 Further studies are needed to explore the environmental risk factors associated with differences in the incidence of psychosis in low and middle-income countries.

**References:**

1. van Os et al. Schizophr Bull. 2014; 40(4):729–36. 2. Jongsma et al. JAMA Psychiatry, in press. 3. Menezes et al. Br J Psychiatry Suppl. 2007:51:102–6.4. Mulè et al. Soc Psychiatry Psychiatr Epidemiol. 2017;52(2):155-62 5. Kirkibride et al. Arch Gen Psychiatry. 2006;63(3):250–8.6. Kirkbride et al. Am J Psychiatry. 2017;174(2):143–53.

